# The Chance of Permanent Cure for Micro- and Macroprolactinomas, Medication or Surgery? A Systematic Review and Meta-Analysis

**DOI:** 10.3389/fendo.2018.00636

**Published:** 2018-10-25

**Authors:** Qianquan Ma, Jun Su, Ying Li, Jiaxing Wang, Wenyong Long, Mei Luo, Qing Liu

**Affiliations:** ^1^Department of Neurosurgery in Xiangya Hospital, Central South University, Changsha, China; ^2^Department of Ophthalmology, Emory University, Atlanta, GA, United States

**Keywords:** microprolactinoma, macroprolactinoma, dopamine agonist, surgery, long-term remission rate

## Abstract

**Background:** This meta-analysis aims to evaluate the long-term efficacy of medication treatment vs. surgery treatment in patients with prolactinomas.

**Methods:** An electronic literature search was performed using MEDLINE, EMBASE and Web of Science databases for studies dated before July in 2018. Patients with prolactinomas received primary dopamine agonists (DAs) treatment or primary surgical interventions were included in this study. A systematic review and meta-analysis were performed in pertinent studies meeting eligible criteria. The clinical outcome was measured by the long-term remission rate of prolactin (PRL) in each cohort. The pooled data was analyzed according to a random effect model.

**Results:** Thirteen publications with total 809 patients were included in the final meta-analysis. In the overall patients with prolactinomas, long-term remission rate was achieved in 88% patients treated with surgeries and in 52% patients treated with DAs (*P* = 0.001). The long-term remission rates in surgery cohort were also significantly higher than medication cohort in both microprolactinomas and macroprolactinomas (91 vs. 60%, *P* = 0.002; 77 vs. 43%, *P* = 0.003).

**Conclusions:** Patients with prolactinomas, especially microprolactinomas, can consider transsphenoidal surgery as an alternative first-line treatment strategy. After receiving primary surgical intervention, administration of DAs should be considered based on the postoperative PRL level to achieve the best long-term remission rate.

## Introduction

Prolactinomas, one of the pituitary adenoma that synthesize prolactin (PRL), account for up to 45% of all pituitary adenomas and are highly associated with symptoms according to hyperprolactinemia in clinical practice (1). In patients with prolactinomas, the most common symptoms are galactorrhea or menstrual disorders in women as well as loss of libido and erectile dysfunction in men (2). According to the size of tumor, prolactinomas are classified as microprolactinoma (<10 mm diameter) or macroprolactinoma (≥10 mm diameter). Microprolactinomas exhibit relatively low secretion of PRL and better prognosis while macroprolactinomas are more often associated with particular difficulties in management due to their high secretion of PRL and aggressive biological behaviors (3, 4).

In clinical practice, dopamine agonists (DAs) are recommended as first-line therapy for patients with prolactinomas to control tumor volume, normalize PRL secretion, alleviate neurologic symptoms and restore normal functions of pituitary (5–7). Surgical treatments are considered as second-line therapy only for patients who cannot tolerate high doses of DAs or who are not responsive to administration of DAs (5). Long-term medication treatments in the form of DAs, including bromocriptine (BRC) and cabergoline (CAB), are highly efficacious to inhibit proliferation of tumor in most cases (8). However, there are many patients who are refractory or intolerant to medical therapies. Relapse of hyperprolactinemia following withdrawal of drug treatment has been observed in a number of studies (9–11). Worse still, adverse effects of DAs on heart valves have also aroused much attention most recently (12, 13). So far, surgical treatments are chosen as first-line therapies only in special conditions like pituitary tumor apoplexy, acute visual deterioration, cranial hypertension or after considering patient preference (14, 15). Based on the fact that modern neurosurgical techniques for pituitary approaches have been remarkably developed over the last decades, especially the development of endoscopic transsphenoidal surgery, normalized PRL level and restored gonadal function following complete resection of tumor have been shown in emerging cases with infrequent severe complications (16–18).

Although it is well-accepted that prolactinomas respond well to DAs treatment and normalization of PRL level can be achieved in the majority of patients during long time intervention, recurrence of hyperprolactinemia in high percentage of patients had been observed after withdrawal of drugs in different studies. Pursuing best prognosis for patients with prolactinomas, the optimum therapeutic strategies are still under discussion and might represent difficulties to clinicians. To give evidence-based recommendations for clinical workers we conducted a meta-analyses to compare the efficacy in long-term normalization of PRL between primary medical treatment and primary surgical treatment in patients with prolactinomas. Our aim is to provide a chance of long-term remission for microprolactinoma and macroprolactinoma.

## Methods

This article was written followed the Preferred Reporting Items for Systematic Reviews and Meta-Analyses (PRISMA) guidelines (19).

### Eligibility criteria

1. Patients with certain diagnosis of microprolactinoma or macroprolactinoma. No any restrictions on age and gender.
2. Patients received either DAs treatments (limited to BRC and CAB) or surgical treatments with various approaches as first-line therapy were included. Patients who had DAs treatment before surgery were excluded from surgery group. Patients who had radio or surgical treatment before DAs intervention were excluded from medication group.
3. Medication group:
  1. Duration time of treatment was at least 2 years and normalization of PRL must be confirmed by solid data during treatment (5).
  2. Patient follow-up period was at least 12 months after drug withdrawal. Patients who became pregnant during this period were excluded. Remission rate after DAs withdrawal should be reported or can be calculated.
  3. Extra information such as gender, age, mean dose of DAs, and mean PRL before treatment should be provided.
  Surgery group:
  1. Patient follow-up period was at least 12 months after surgery. Long-term remission rate should be reported or calculated.
  2. DAs maintenance after surgery should be mentioned if needed.
  3. Extra information such as gender, age, mean dose of DAs, and mean PRL before surgery should be provided. It's better if the short term remission rate immediately after surgery was reported.
4. We summarized all types of studies including case reports with at least 3 subjects.

### Search strategy

The following databases were searched to identify articles addressing prolactinomas treated by medical or surgical strategies: Medline/PubMed, EMBASE/Ovid, and Web of Science. Searches were performed in July 2018, using “prolactinoma,” “prolactin-secreting pituitary adenoma,” “hyperprolactinemia,” “medical treatment,” “dopamine agonist,” and “surgical treatment” as key phrases in various combinations. Search strategy was modified to suit each database. We didn't impose any language restrictions. We also conducted a manual search of reference lists from each targeted article to acquire additional related studies. In order to identify ongoing relevant clinical trials, extra search was also carried on ClinicalTrials.gov.

### Study extraction

Qianquan Ma (Primary reviewer) and Jun Su (secondary reviewer) independently screened the titles and abstracts for each paper found in the search procedure and obtained full-text versions of all potentially eligible studies. Once full-text articles had been retrieved, reviewers checked the studies again and applied eligibility criteria to further exclude papers. All disagreements received final consensus after several serious discussions between reviewers. In cases where studies provided limited information on the intervention or post-treatment outcome, authors were contacted to provide data in detail. Full data extraction in data extraction sheet was completed after reviewers independently identified cases from every targeted article and reached final agreement. Data extraction form contained the following information about enrolled patients, therapeutic interventions, clinical outcomes and study quality measures.

### Statistical analysis

Data are presented as frequencies, as the mean ± standard deviation or as the median (range). The 1-sample Kolmogorov–Smirnov test was performed to examine whether the samples distributed normally. Differences in gender, age, mean PRL level before treatment and long-term remission rate were estimated by Mann–Whitney-*U* test. Forest plots were performed using the software R version 3.4.0 and package “Meta.” All other statistical analyses were performed using commercial statistical software (IBM SPSS Statistics 24.0). A value of *P* < 0.05 was considered statistically significant.

## Results

### Search results

**Figure 1:**
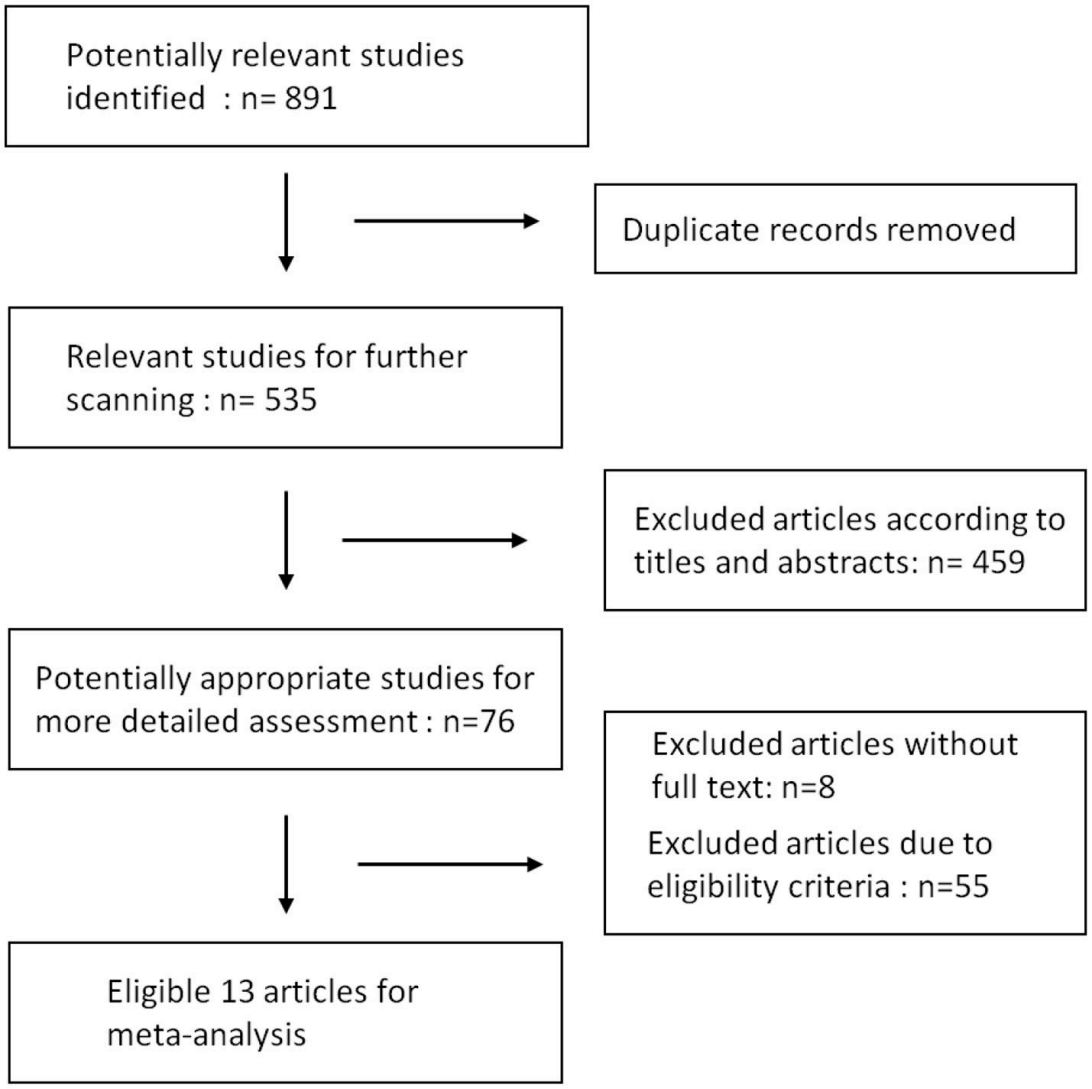
Flowchart of literature search and study selection.

Results of our research strategy are shown in Figure 1. Potentially relevant publications were identified through the literature search from multiple databases before July, 2018. Based on the quick scan of titles and abstracts of articles, we identified 76 articles as potential targets for further full-text analysis. There were 8 articles without full text and most of them were recorded in EMBASE database exclusively. After assessment with eligibility criteria in detail, 13 publications with total 809 patients were included in the final meta-analysis (9–11, 20–29). Details of the 13 articles are summarized in Table 1.

**Table 1:** Characteristics of studies included in the meta-analysis.

**Author, year of publication**	**No. of patients**	**Micro/ Macro**	**Mean follow up time (months)**	Mean age(year)^*^	**Male/Female $**	**First-line treatment**	**Mean PRL before Treatment (ng/ml) ¥**	**Normalization of PRL in therapy (Medical cohort)**	**Short term remission rate (Surgical cohort)**	**Long-term remission rate**
**MEDICATION COHORT**										
(28)	89	Micro:30	>12	33	1/29	CAB or BRC	219.2 ± 460.6	Y (5.0 ± 5.1)	NA	13 (43.3%)
		Macro:59		34	26/33		936.5 ± 1947	Y (4.2 ± 4.6)		25 (42.4%)
(29)	50	Micro:41	>12	35.1	5/45	CAB or BRC	93.3	Y	NA	32 (78.0%)
		Macro:9					521	Y		4 (44.4%)
(25)	74	Micro:56	>12	42.4 ± 9.9	6/50	CAB	113.5 ± 54.12	Y (7.3 ± 4.3)	NA	31 (55.3%)
		Macro:18		70 ± 10.9	13/5		258.9 ± 211.3	Y (13.4 ± 14.1)		9 (50.0%)
(11)	67	Micro:23	>12	34.3 ± 11.2	2/21	CAB or BRC	142 ± 35 ~ 207 ± 112	Y	NA	15 (65.2%)
		Macro:44			15/29		938 ± 1369 ~ 2902 ± 4591	Y		16 (36.4%)
(10)	42	Micro:31	>12	44	5/26	CAB	73.0	Y (3.6)	NA	15 (48.4%)
		Macro:11		54	7/4		310	Y (1.9)		5 (45.5%)
(9)	194	Micro:115	>12	32	12/103	CAB	222.5	Y	NA	76 (66.1%)
		Macro:79		44	36/43		1261.8	Y		37 (46.9%)
(20)	89	Micro: 89	>12	32.7	5/84	CAB or BRC	100.9	Y	NA	32 (36.0%)
**SURGERY COHORT** #										
(27)	71	Micro:41 Macro:30	>12	33.3 ± 10	0/71	TS	105	NA	67%	90%
							303	NA	61%	81%
(26)	15	Micro:5 Macro:10	>12	42.8 ± 13	5/0	TS	220	NA	80%	4 (80.0%)
					10/0		3130	NA	30%	8 (80.0%)
(24)	65	Micro:54 Macro:11	>12	37.11	16/38	TS	170.07 ± 120.16	NA	ND	53 (98.1%)
					9/2		266.05 ± 190.77			7 (63.6%)
(23)	10	Micro:10	>12	31.0 ± 8.2	0/10	TS	108.9	NA	ND	9 (90.0%)
(22)	9	Micro:2	>12	19	0/2	TS	123.50	NA	100%	2 (100%)
		Macro:7		50	5/2		3171.28	NA	42.9%	5 (71.4%)
(21)	34	Micro:24	>12	30.2	4/20	TS	106.5	NA	91.7%	22 (91.7%)
		Macro:10		32.6	0/10		273	NA	100%	8 (80.0%)

Y, yes; ND, no data; NA, not applicable; TS, Transsphenoidal surgery;

# *Part of patients needed postoperative administration of DAs for long-term normalization of PRL*.

*Potential sources of heterogeneity between medication and surgery cohorts were estimated*.

* P = 0.534;

$ *P = 0.836; ¥: Micro: P = 0.485; Macro: P = 1.000*.

### Clinical outcomes of interventions

To evaluate the potential sources of heterogeneity, we conducted Mann–Whitney-*U* test for factors between the medication cohort and surgery cohort. No statistical differences were found in age, gender, and pre-treatment PRL levels (Table 1).

### Medication vs. surgery as first-line treatment on the long-term remission rate of all prolactinomas

**Figure 2:**
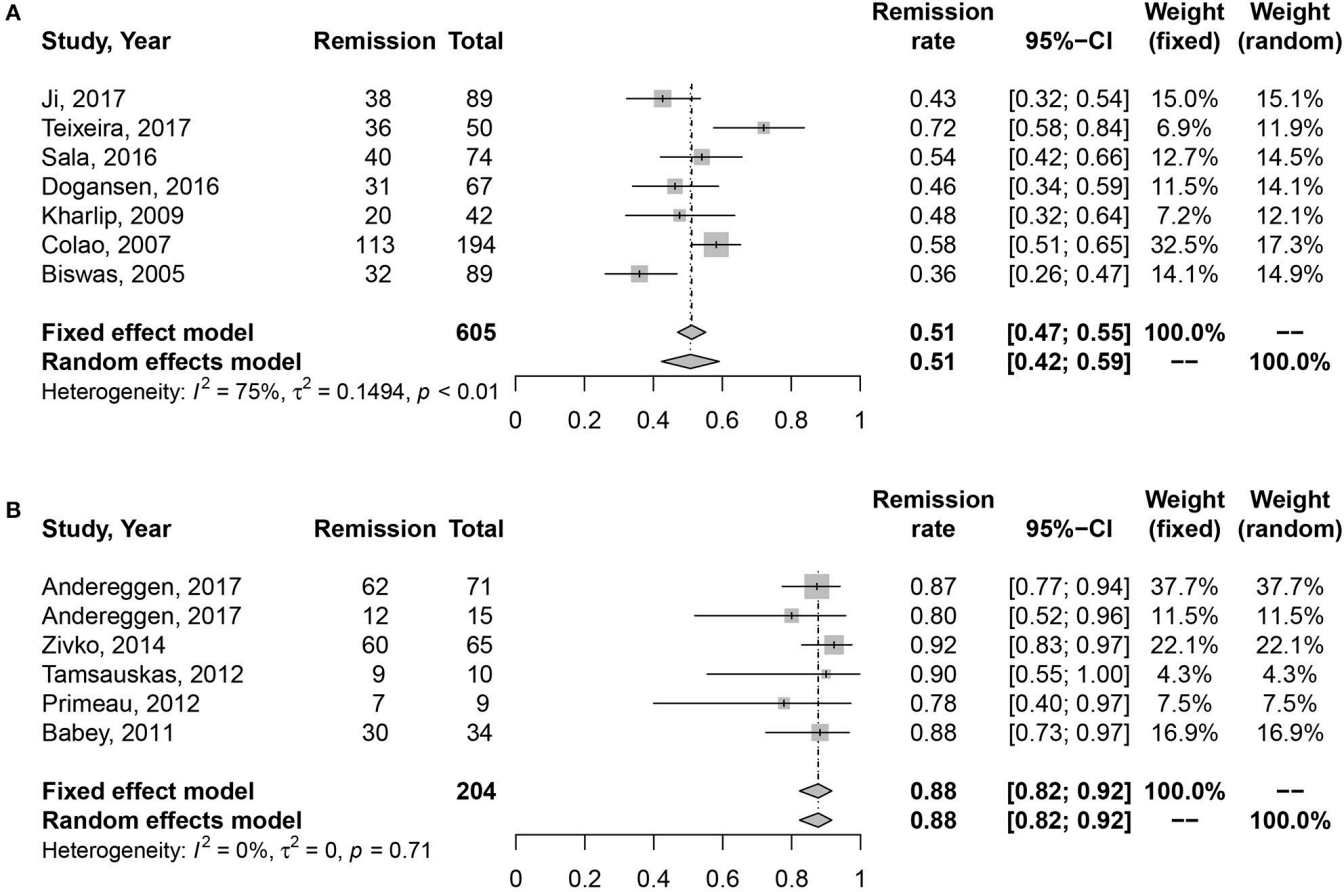
Forest plots describing effects of medication vs. surgery as first-line treatment on the long-term remission rate of all prolactinomas. **(A)** Long-term remission rate in medication cohort for all prolactinomas. **(B)** Long-term remission rate in surgery cohort for all prolactinomas.

Due to the high heterogeneity of effect size in medication cohort (*I*2 = 70%, *P* < 0.01), random effects model was used for meta-analysis. Results are shown in Figure 2. The long-term remission rate was 52% (95% CI: 0.43–0.61) in patients treated with DAs when compared with 88% (95% CI: 0.82–0.92). A significant difference was found between the two groups (*P* = 0.001), Table 2.

**Table 2:** Differences in long-term remission rate of PRL between medication and surgery.

**Long-term remission rate**	**Intervention**		***P***
	**Medication %**	**Surgery %**	
All	51	88	0.001
Micro	56	91	0.001
Macro	44	77	0.004

### Medication vs. surgery as first-line treatment on the long-term remission rate of microprolactinomas

**Figure 3:**
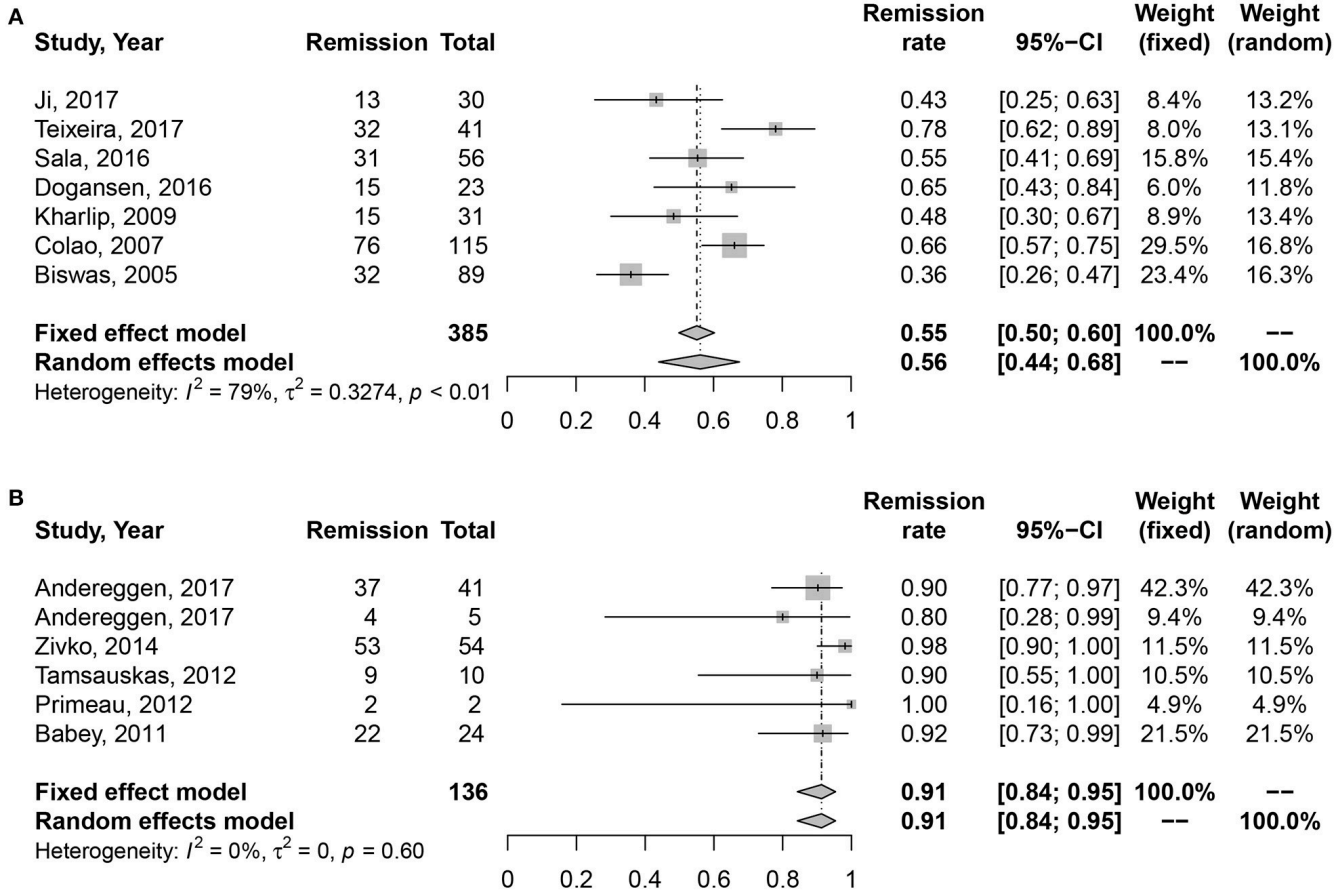
Forest plots describing effects of medication vs. surgery as first-line treatment on the long-term remission rate of microprolactinomas. **(A)** Long-term remission rate in medication cohort for microprolactinomas. **(B)** Long-term remission rate in surgery cohort for microprolactinomas.

High heterogeneity was also detected in medication cohort (*I*2 = 61%, *P* = 0.03), so we chose random effects model for analysis. The results in Figure 3 indicated higher long-term remission rate in surgery cohort, 91% (95% CI: 0.84–0.95) than DAs cohort, 60% (95% CI: 0.50–0.69). Meanwhile, significant difference was shown in Table 2 (*P* = 0.002).

### Medication vs. surgery as first-line treatment on the long-term remission rate of macroprolactinomas

**Figure 4:**
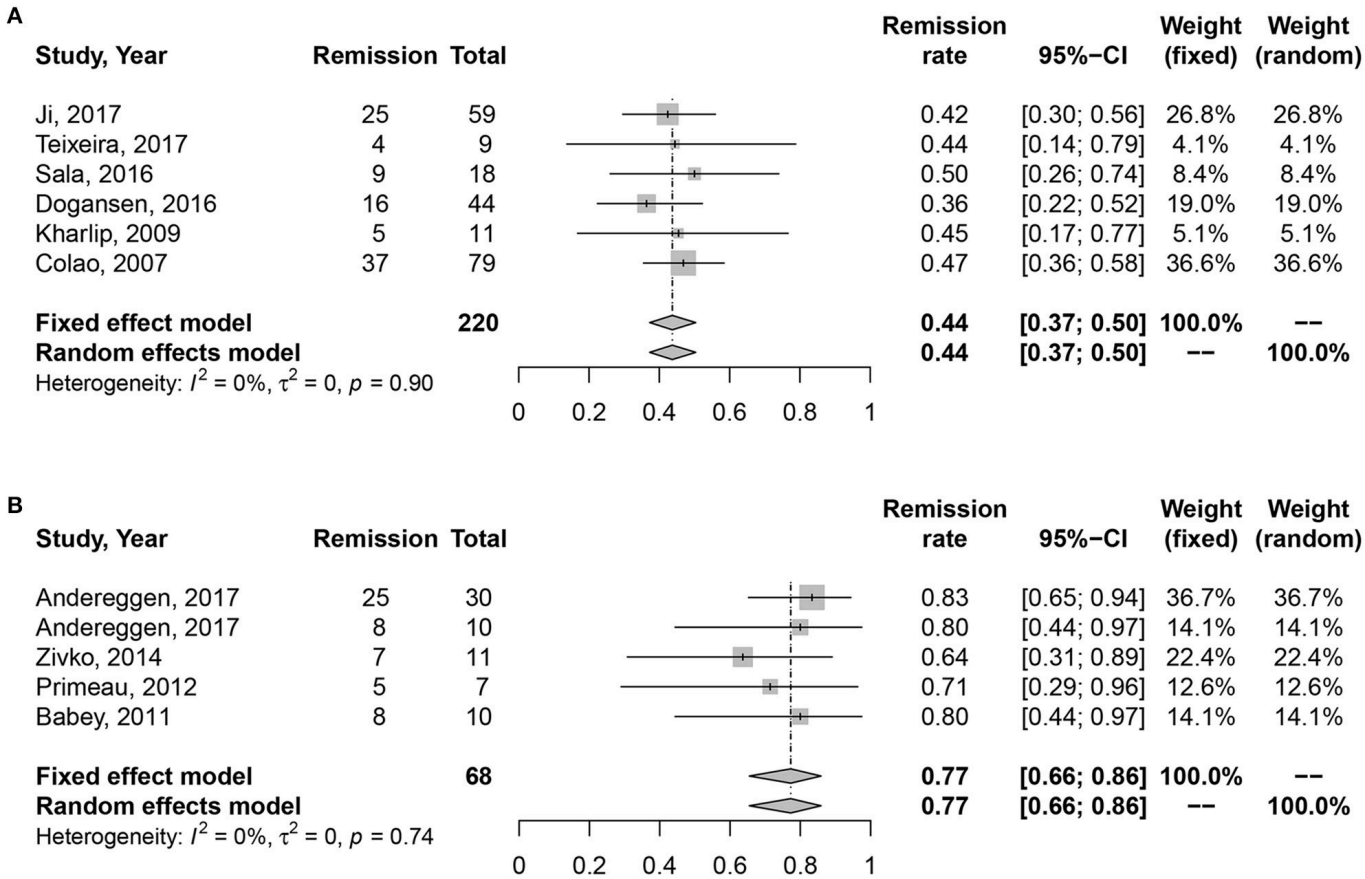
Forest plots describing effects of medication vs. surgery as first-line treatment on the long-term remission rate of macroprolactinomas. **(A)** Long-term remission rate in medication cohort for macroprolactinomas. **(B)** Long-term remission rate in surgery cohort for macroprolactinomas.

No heterogeneity was found in studies (*I*2 = 0%, *P* = 0.45; *I*2 = 0%, *P* = 0.74). Random effect model was analyzed for this research. Data in Figure 4 showed consistent results with all and microprolactinomas. Better prognosis were identified in surgery cohort, 77% (95% CI: 0.66–0.86) than medication cohort, 43% (95% CI: 0.36–0.49). There is also significant difference shown in Table 2 (*P* = 0.003).

### Risk of bias

The influence of single study on the overall risk was assessed graphically using funnel plots. The funnel-shaped with the apex near the symmetry, which indicated that no study had a large impact on the results. However, the relatively low numbers of publications in surgical cohort may lead to a risk of bias. The unavailable studies without full-text might be another reason for risk of bias.

## Discussion

The most commonly recommended treatments for prolactinomas are CAB and BRC. Both of the two drugs are characterized to activate dopamine receptor expressed on prolactinoma cells, therefore cause cell death, decrease cellular metabolism and inhibit PRL production and secretion (30, 31). It had been demonstrated that CAB is more effective and better tolerated than BRC with less frequency of dosing (32, 33). However, in patients with long-term DAs treatment, except the well-known adverse effects such as headache, dizziness, nausea, and vomiting, the concern of increased risk of cardiac valve abnormalities has also arisen recently (34, 35). Despite that the administration of DAs had been applied in patients with prolactinomas with great success, quite a few patients were reported to experience the recurrence of hyperprolactinemia after withdrawal of drugs, even if they met strict discontinuation criteria during the duration of treatment (9, 10, 25). Obtaining sufficient DAs treatment over 2 years with normalized level of PRL and obvious reduction of tumor mass (50% or more), there are still possibilities for the hyperprolactinemia recurrence after drug withdrawal, which suggests medication treatment cannot guarantee a long-term remission even in responders to DAs therapies. So far, pituitary surgery is accepted as second-line treatment in non-responders to medical therapy or in those who cannot tolerate side effects of DAs. It is also regarded as first-line therapy in special conditions such as intratumoral hemorrhage or apoplexy (36). Given the advanced growth of modern neurosurgery, especially the development of endoscopic transsphenoidal techniques, greater extent of resection and improved safety can be achieved by the larger field of visualization during operations. The endoscopic approach offers more effective and safer resection of tumor tissue with the superior close-up view and enlarged vision inside surgical area. Thereby, more normal nervous tissue can be identified and preserved during manipulation (37). Higher rates of hormone restoration and visual improvement, and lower incidence of postoperative complications all indicated that effective modern pituitary surgery may be an alternative strategy for the management of prolactinomas (21, 38). Besides, evidence showed that surgical cure rates are lower in patients who received DAs treatment prior to operation, possibly due to the drug-induced tumor fibrosis (39, 40). From this perspective, we have reasons to consider, between medication and surgery, which is the optimal choice for prolactinomas, particularly for patients with microprolactinomas and low PRL level.

To directly compare the long-term remission rates of medication treatment vs. surgery treatment, we conducted a meta-analysis in patients received surgery as first-line therapy and in patients treated with DAs as first-line therapy. In medication cohort, we selected patients who met the criteria of drug withdrawal, indicating they were sensitive to DAs and achieved normalization of PRL level in treatment procedures. Therefore, patients with resistance or intolerance to drug therapies were excluded from this study. Our aim in this study was to estimate the prognosis in patients accepted different interventions, so long-term remission rate was the exclusive measurement. Surprisingly, results from this pooled analysis showed long-term remission rates of surgery cohort were significantly higher not only in overall prolactinomas, but also in microprolactinomas and macroprolactinomas, respectively. In our study, the remission rate of 91% is slightly higher than previously reported surgical remission rates from 82 to 86% for patients with microprolactinomas (41–44). The remission rate of 77% is almost similar to the reported data for patients with macroprolactinomas, ranging from 48 to 76% (45–47). Neither study in surgery cohort showed mortality. Transient diabetes insipidus, cerebrospinal fluid rhinorrhea, visual loss and paresis of oculomotor and abducens nerve were the major morbidities in low frequencies. Postoperative hypocorticism in few patients were also recorded in some studies. Although the long-term clinical outcome of primarily surgical therapies was significantly better than primarily DAs therapies, most studies in our surgery cohort mentioned the unignorable contribution of DA treatment for patients with uncontrolled PRL level after operation, which demonstrated the necessity of multi-therapeutic strategy in some cases. Considering nearly 10–20% patients do not respond to DAs treatment in terms of PRL normalization or are intolerant of the side effects (48, 49), and this portion of patients was excluded from our medication cohort, the total clinical remission rates in patients treated with DAs as first-line therapies must be even lower than the results in our study. Moreover, another research proved the connection of high surgical remission rate with preoperative RRL levels. Ninety-two percent of the patients with preoperative PRL levels < 100 ng/ml and 75% of the patients with preoperative PRL levels between 101 and 200 ng/ml experienced promising clinical prognosis, while only 37% of patients with preoperative PRL levels >200 ng/ml achieved successful surgical outcomes (50). In this regard, it is reasonable for experienced and handy neurosurgeon to recommend surgical management as primary option to achieve better long-term prognosis, especially in patients with microprolactinomas or low preoperative PRL level. DAs maintenance or not should depend on the postoperative level of PRL.

Recently, some studies also performed analysis to compare the impact of two strategies on the overall cost of treatment and quality of life for patients with prolactinomas. Data on the cost-effectiveness analysis revealed that medication was more costly and less effective than surgery in young patients with microprolactinomas with life expectancy >10 years (51). Another study further accomplished sensitivity analysis and proved surgery was a more cost-effective treatment for prolactinomas than medical management or a wide range of characteristics of patients (52). Meanwhile, other studies showed that surgically treated patients had a similar quality of life compared to healthy controls (53), while the quality of life is impaired in DAs treated patients, specifically due to increased anxiety and depression (54, 55).

## Limitations

Limitation of this meta-analysis should be mentioned. First, because few studies have been published focusing on the first-line surgical treatment of prolactinomas, the number of patients in surgery cohort was far less than medication cohort. Even though the long-term remission rates are much higher in patients treated with surgery, there are possibilities for the existence of bias due to the relatively small sample size, especially in subgroup of macroprolactinomas. Publication bias may also exist from the original studies. Second, details of postoperative DAs administration in surgical cohort were not clear, so it's impossible for us to compare the exact dose and duration time in patients receiving DAs after surgeries and in patients accepting DAs as first-line treatment. These issues highlight the importance to evaluate DA treatment vs. transsphenoidal surgery with respect to long-term remission rates, drug adverse effects and surgical complications in a randomized clinical trial among patients with prolactinomas. Third, as we excluded the group of patients who are intolerant or resistant to DAs therapies in medical cohort, the long-term remission rates in our study cannot represent the general remission rates. General remission rates in patients with drug treatment should be even lower than our study. Fourth, although we considered surgical intervention can be first option for patients, there is a lack of standardization of surgical indication in our paper. Further investigations are warranted to identify these kinds of information in detail. Fifth, due to the limitation of detail information, prolactinomas were only divided into subgroups of micro and macro. The definition of giant prolactinomas was not applied in this study, resulting in the lack of full understanding of this kind of invasive prolactinomas. Further investigations are needed to illustrate best therapeutic strategy against giant prolactinomas.

## Conclusion

Taken together, modern transsphenoidal surgery may be optimal in terms of long-term remission rate and thus seems like a reasonable alternative strategy especially in patients with microprolactinomas. Additionally, surgical interventions are also reported to benefit the economic costs and quality of life of patients. After receiving first-line surgical treatment, administration of DAs should be considered based on the postoperative PRL level to achieve the best clinical outcomes.

## Author contributions

QM, JS, and QL conceived and designed the experiments. QM, JS, YL, and ML performed the experiments. YL, JW, and QM analyzed the data. QM, JW, JS, and WL wrote the manuscript. QL supervised the entire work. QM, JS, YL, JW, WL, ML, and QL provided final approval for the version to be published.

### Conflict of interest statement

The authors declare that the research was conducted in the absence of any commercial or financial relationships that could be construed as a potential conflict of interest.
